# Supplementary figures and images for: Brain Extract of Subacute Traumatic Brain Injury Promotes the Neuronal Differentiation of Human Neural Stem Cells via Autophagy
Source: J Clin Med. 2022 May 11;11(10):2709. doi: 10.3390/jcm11102709 (PMC9145659; doi:10.3390/jcm11102709)

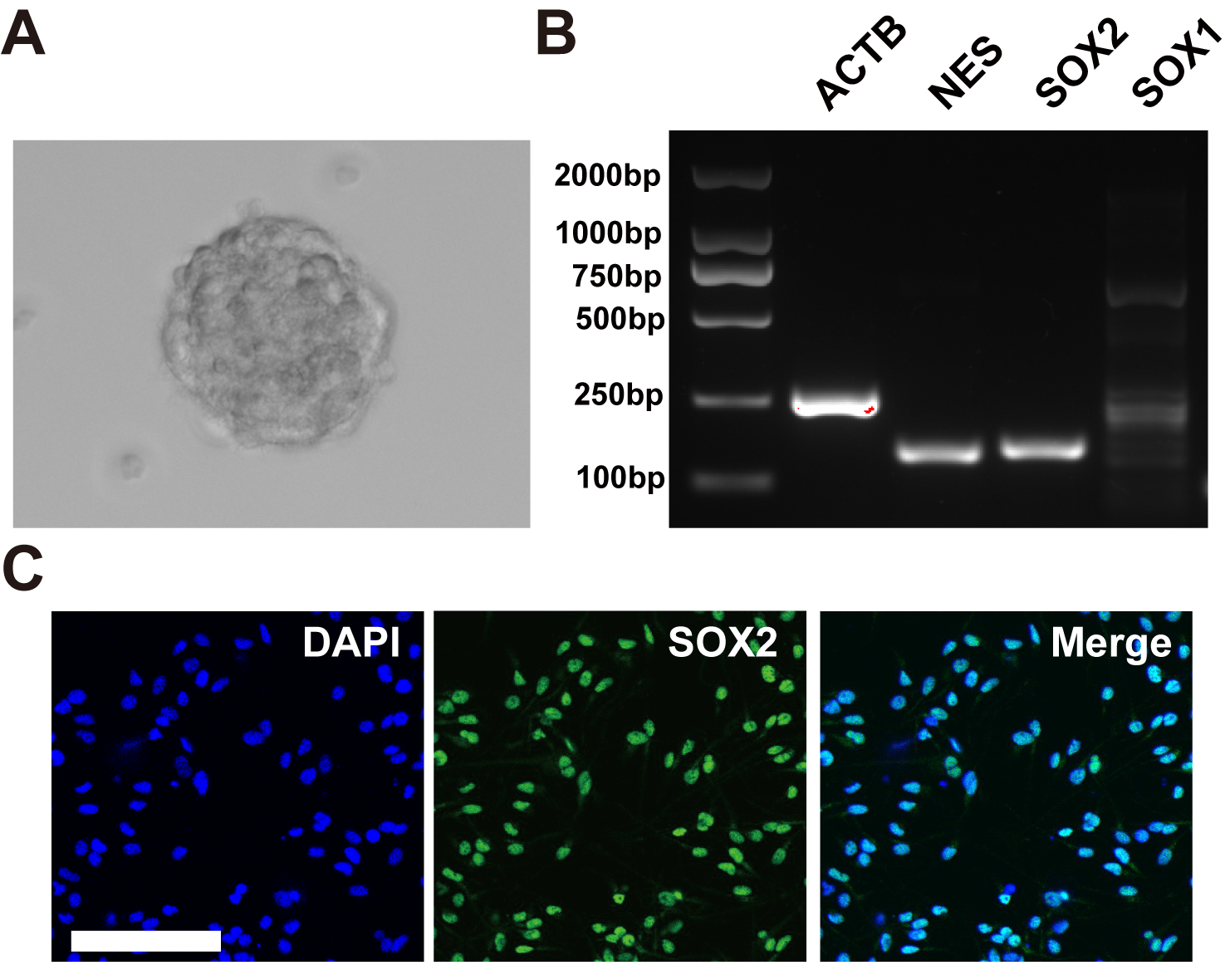

Supplement: Supplementary file 1 [file jcm-11-02709-s001.zip › Figure S1.tif]

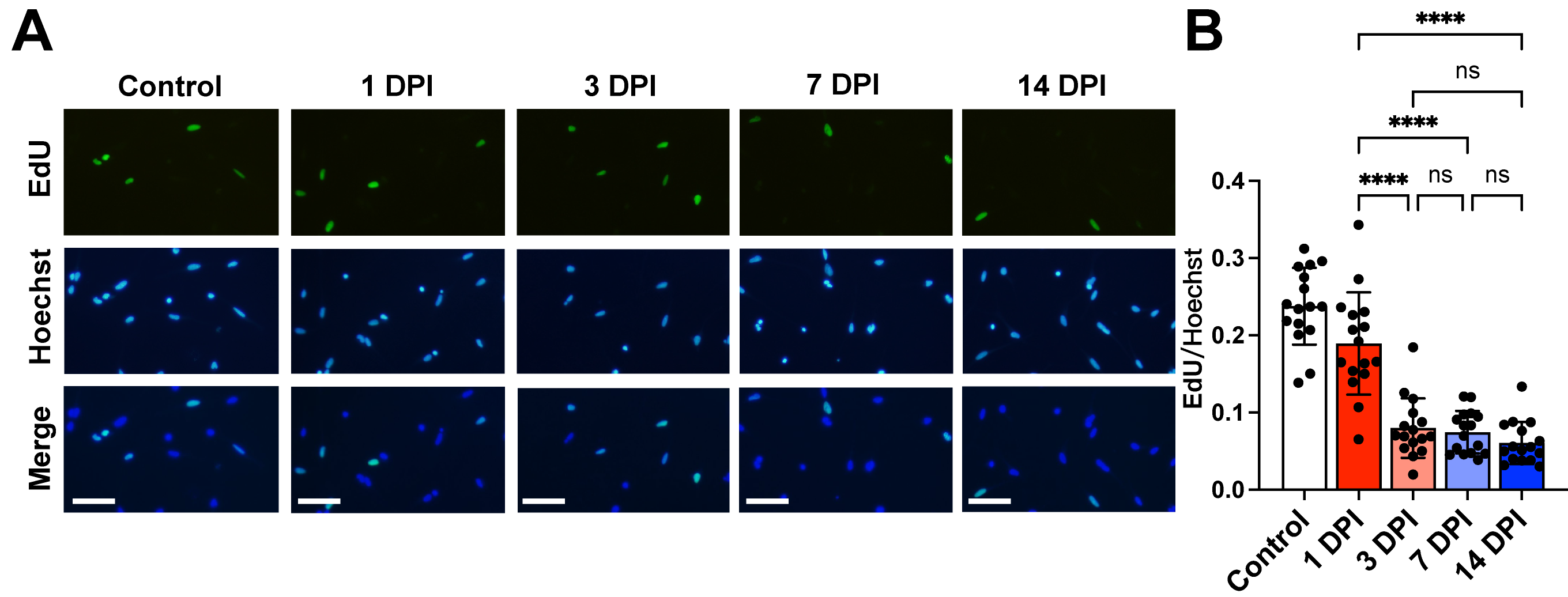

Supplement: Supplementary file 1 [file jcm-11-02709-s001.zip › Figure S2.tif]

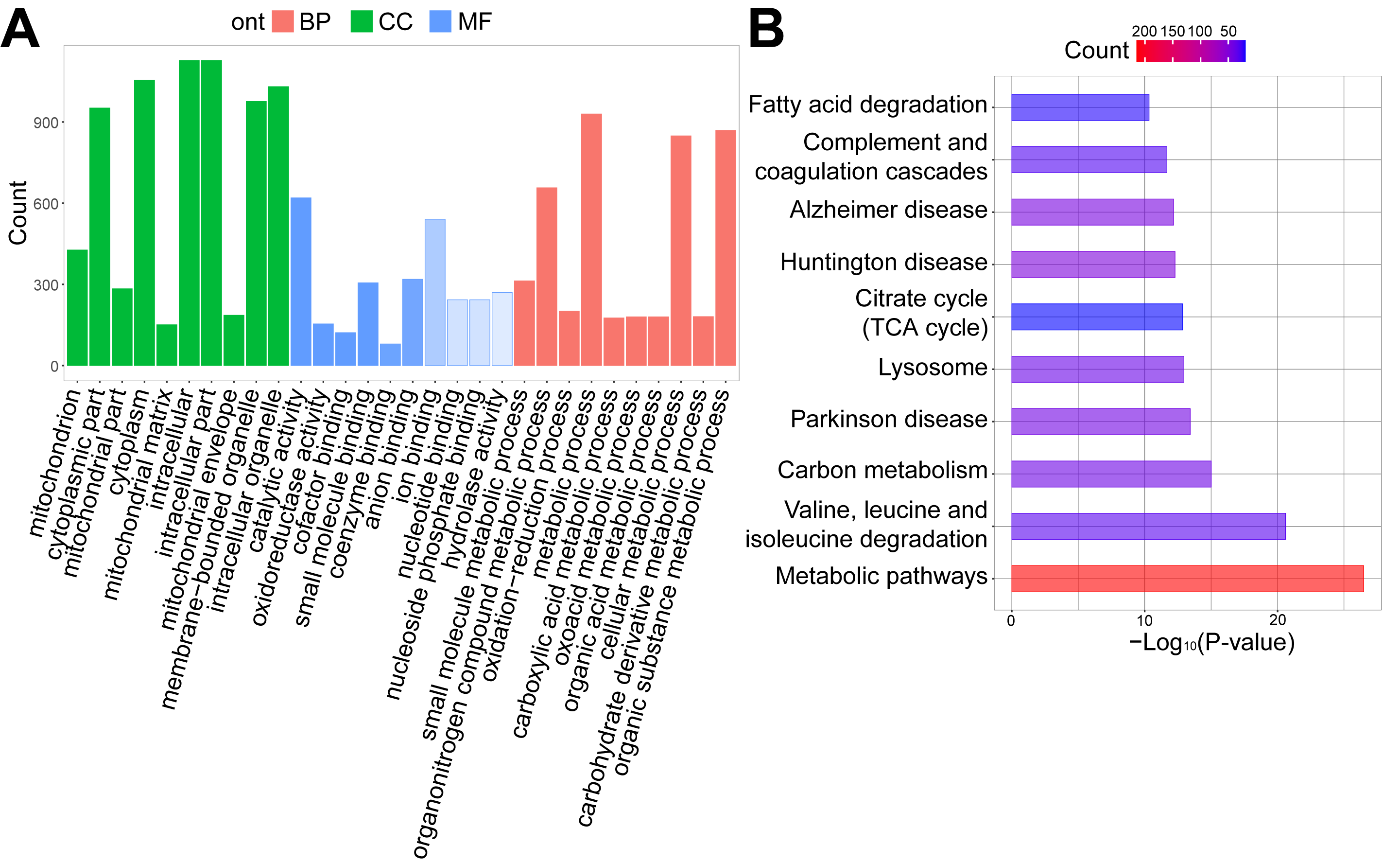

Supplement: Supplementary file 1 [file jcm-11-02709-s001.zip › Figure S3.tif]
